# Artefacts of PET/CT images

**DOI:** 10.2349/biij.2.4.e60

**Published:** 2006-10-01

**Authors:** C Pettinato, C Nanni, M Farsad, P Castellucci, A Sarnelli, S Civollani, R Franchi, S Fanti, M Marengo, C Bergamini

**Affiliations:** 1 Health Physics Department, Azienda Ospedaliero Universitaria S. Orsola Malpighi, Bologna, Italy; 2 Nuclear Medicine Division, Azienda Ospedaliero Universitaria S. Orsola Malpighi, Bologna, Italy

**Keywords:** PET/CT, artefacts, attenuation correction

## Abstract

Positron emission tomography (PET) is a non-invasive imaging modality, which is clinically widely used both for diagnosis and accessing therapy response in oncology, cardiology and neurology.

Fusing PET and CT images in a single dataset would be useful for physicians who could read the functional and the anatomical aspects of a disease in a single shot.

The use of fusion software has been replaced in the last few years by integrated PET/CT systems, which combine a PET and a CT scanner in the same gantry. CT images have the double function to correct PET images for attenuation and can fuse with PET for a better visualization and localization of lesions. The use of CT for attenuation correction yields several advantages in terms of accuracy and patient comfort, but can also introduce several artefacts on PET-corrected images.

PET/CT image artefacts are due primarily to metallic implants, respiratory motion, use of contrast media and image truncation. This paper reviews different types artefacts and their correction methods.

PET/CT improves image quality and image accuracy. However, to avoid possible pitfalls the simultaneous display of both Computed Tomography Attenuation Corrected (CTAC) and non corrected PET images, side by side with CT images is strongly recommended.

## INTRODUCTION

Positron emission tomography (PET) is a non-invasive imaging modality, which is clinically widely used both for diagnosis and accessing therapy response in oncology, cardiology and neurology [1-3].

Because of its very high sensitivity it is an excellent tool to recognise malignant nodules and lesions earlier than their anatomical compromising. The lack of anatomic information in PET images can be compensated by other complementary imaging techniques such as CT or MRI read side by side. Several methods have been developed to register and fuse PET and CT data acquired on separate systems [4-5]. The major problems related with image fusion are the different formats of images of the two datasets and the need to use external markers, visible with both modalities, to be sure to have a good match among corresponding images.

The ideal condition for image fusion is to have the two datasets acquired closely sequentially on the same system [6-7].

It has been well established that the fusion of PET and CT provides information exceeding the sum derivable from the two modalities treated separately [8-17].

The advantages of PET/CT over PET are:

1. Faster and less noisy attenuation correction maps
2. Better diagnostic accuracy especially in disease staging
3. Better ability to identify and localise lesions
4. Shorter transmission acquisition time with a consequent better comfort for the patient and less probability of patient motion.

This paper describes all different artefacts that can be caused by the use of a combined PET/CT system and that can affect the accuracy of PET-corrected images [18-19].

## PET/CT SCANNER DESIGN

A PET/CT scanner combines PET and CT technology in the same gantry. The patient, lying on the table, undergoes CT and the PET scan sequentially.

The first PET/CT system, developed and installed at the University of Pittsburg, was based on the combination of a spiral CT scan (Somatom AR.SP) with a rotating partial ring PET scanner (ECAT ART) [20].

**Figure 1:**
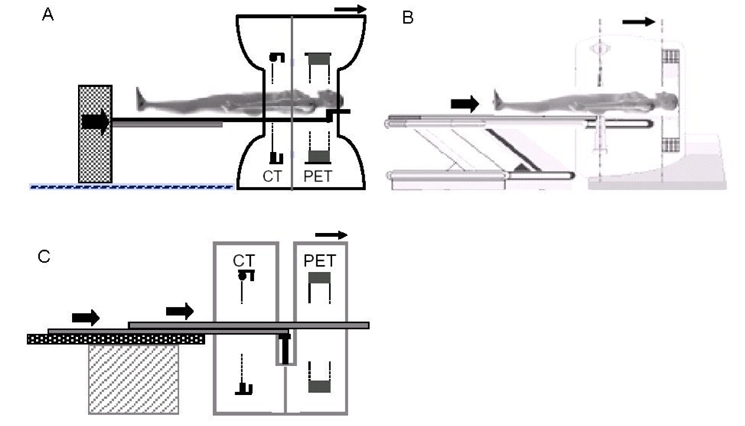
These images show the layouts of the three commercial family systems available on the market: a) Siemens/CTI Biograph, b) GE Healthcare Discovery, c) Philips Gemini.

In all modern commercial systems [21-24] the CT is on the front and the PET is on the back: the patient first undergoes the CT scan and then the PET scan (Figure 1).

No limitations exist on the type of systems employed: the CT can be single or multislice, working in either axial or helical mode while the PET system can use a different crystal material (BGO, LSO, LYSO, GSO). Some PET systems can acquire in either 2D or 3D mode whereas others can only acquire in 3D mode.

## ACQUISITION PROTOCOLS

A PET/CT acquisition protocol has three steps: a) SCOUT acquisition for axial Field of View (FOV) definition, b) CT acquisition, and c) PET acquisition.

Because CT is used mostly to fuse anatomical information to functional PET images and to correct attenuation, low-dose CT protocols can be adopted as a compromise between acceptable image quality and absorbed dose to the patient. This kind of CT images cannot be used on their own for diagnosis.

The common CT protocol uses 100-140 kV and 60-100 mA: the nuclear medicine technologist should modify these values according to the weight of the patient [25]. Additional conservative parameters should be selected for paediatric studies.

The duration of PET scan is about 3-5 minutes/bed position and depends on different factors such as the acquisition mode (2D or 3D), the injected dose and the time between the administration of the activity and the acquisition start time. Because PET image matrix size is 128x128 and CT is 512x512, CT data need to be rebinned to perform image registration and attenuation correction.

## ATTENUATION CORRECTION

In conventional PET, attenuation correction is done using transmission scans acquired with external radioactive sources: most systems use ^68^Ge rods. The transmission acquisition time varies from 2 to 4 minutes/bed position depending on the correction method used (segmented versus measured) [26-27].

The use of CT transmission maps for attenuation correction reduces transmission acquisition time to 1-2 minutes, including SCOUT and whole body CT scans, together with increased accuracy of attenuation coefficients.

Because of the different energy of CT photons compared with the emission photons (about 80 KeV versus 511 KeV) all commercial systems have a scaling algorithm to convert the correction factors from CT to PET [28-29].

All photon attenuation information embedded in the CT data is translated into the PET images because of the attenuation correction. For this reason most of the PET/CT artefacts are related to the CT images and need to be accurately identified to avoid false positive reports.

## IMAGE ARTEFACTS

PET/CT image artefacts are due primarily to metallic implants, respiratory motion, use of contrast media and image truncation. All these artefacts are visible in both CT alone and in CTAC PET images. The artefacts do not appear in uncorrected PET images, so they may be used as control images for testing doubtful findings.

## Metallic implants

The presence of metallic implants, such as dental clogging, dental implants, metallic clips and chemotherapy infusion ports, is visualised by CT images as areas of high density, which cause artefacts on the CT images [30-31]. These high CT numbers correspond to high attenuation coefficients that result in an overcorrection of the PET images, promoting false-positive findings. The uncorrected images can help the nuclear medicine physician to identify these “hot” findings as artefacts.

**Figure 2:**
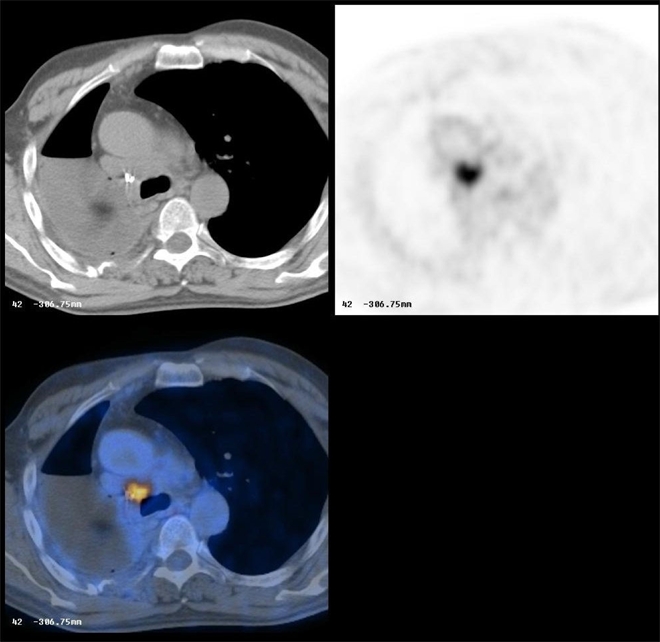
Focal artefact on CTAC PET images due to the presence of a metallic clip.

**Figure 3:**
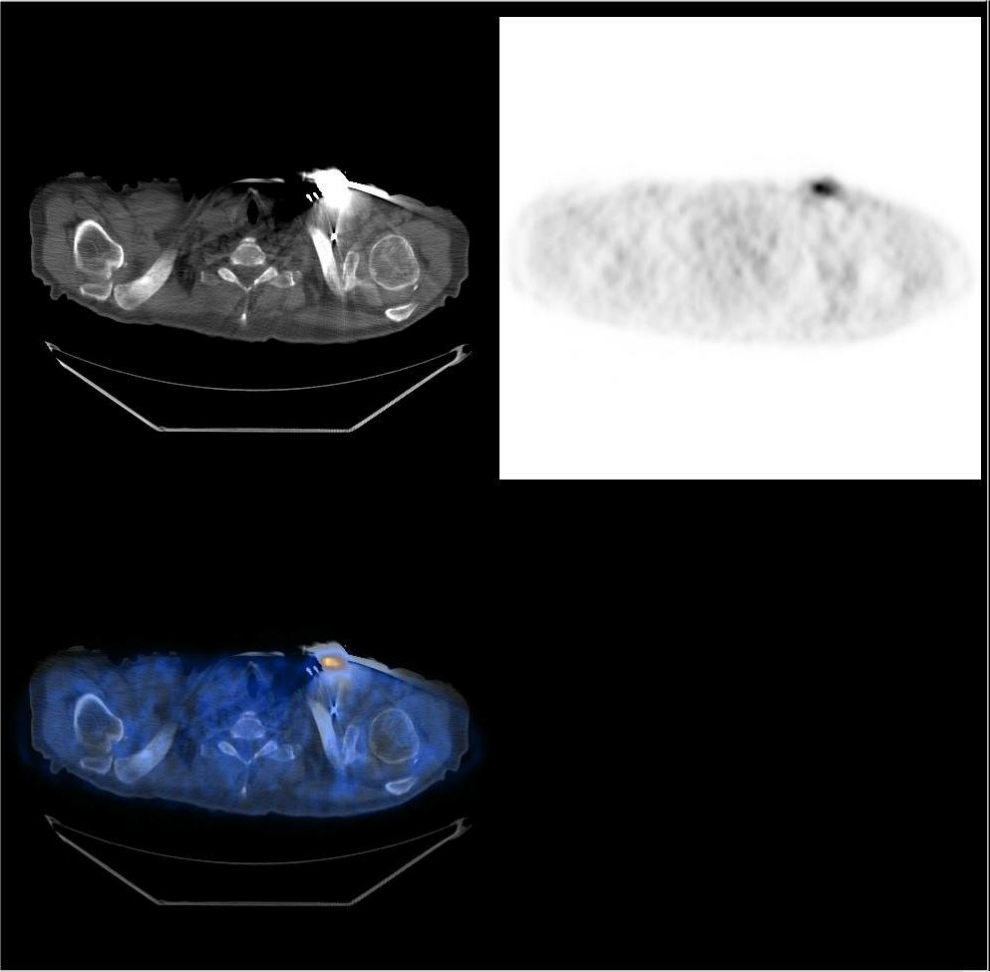
Focal artefact on CTAC PET images due to the presence of a pace maker.

Figure 2 shows a typical artefact due to the presence of a metallic clip; it is very clear the effect of the higher CT correction on the PET images producing a false-positive finding. A similar artefact can be caused by the presence of a pace maker (Figure 3).

If the metallic implant size is sufficiently large (for example, a hip implant), the PET images do not present an artefact because the implant area is characterised by the absence of activity in the prosthetics. Therefore, though the CT-derived attenuation coefficients are high, the corrected and uncorrected images are similar and are visualised as “cold” regions [32-34].

To minimise the presence of artefacts due to metallic implants, the technologist should ask the patient to remove before scanning all metallic objects, such as coins, jewels, metallic buttons, belt buckles, bra with iron inserts. Physicians should highlight in the anamnesis the presence of non-removable metallic implants.

## CT contrast media

To better visualise vessels and soft tissues and to improve CT image quality, intravenous or oral contrast media are often administered to patients. However, the use of these agents can introduce changes into CT numbers similar to metallic implants, affecting the quantitative and qualitative accuracy of CTAC PET images [35-41]. The effect of contrast media artefacts increases with the concentration of the administered agent and depends on its clearance from patient’s body and the time between administration and CT acquisition. In particular, the tissue concentration of oral contrast agents increases over time, so while their use during a PET/CT protocol gives all the benefits related to a better visualization of CT images without a real compromising of CTAC PET images, particular attention should be taken if the patients had undergone a diagnostic CT scan with contrast few hours before the PET/CT scan.

Several correction techniques are presented in the literature [42]. Nehmeh *et al*. [43] propose an interesting method to correct for CTAC PET images. This method is performed by contouring the contrast regions, excluding any body structures; transforming the corresponding linear attenuation coefficients, μ(x, E), of contrast correctly from CT to PET energies; and, finally, reconstructing CTAC PET images with the appropriately scaled attenuation map.

## Respiratory motion

One of the most significant and frequent artefact in PET/CT images is due to respiratory motion during scanning. Although the use of a combined PET/CT scanner allows the registration of the two datasets in the simplest way, respiratory motion results into mismatch between CT and corresponding PET slices [44-46]. Because of the long acquisition time of the PET scan, the patient is allowed to breath normally during both CT and PET acquisitions. Asking the patient to hold the breath during the CT scan, as it’s normally done in diagnostic CT studies, can lead to artefacts because of the certain mismatch between a specific stage of the breath cycle during the CT and the average of many breathing cycles of the PET images. However, even if the patient is usually allowed to breathe normally during the whole PET/CT study, because of the fast CT, the diaphragm is visualised in a single position that is different from the mean position of PET images or in the course of respiratory motion.

**Figure 4:**
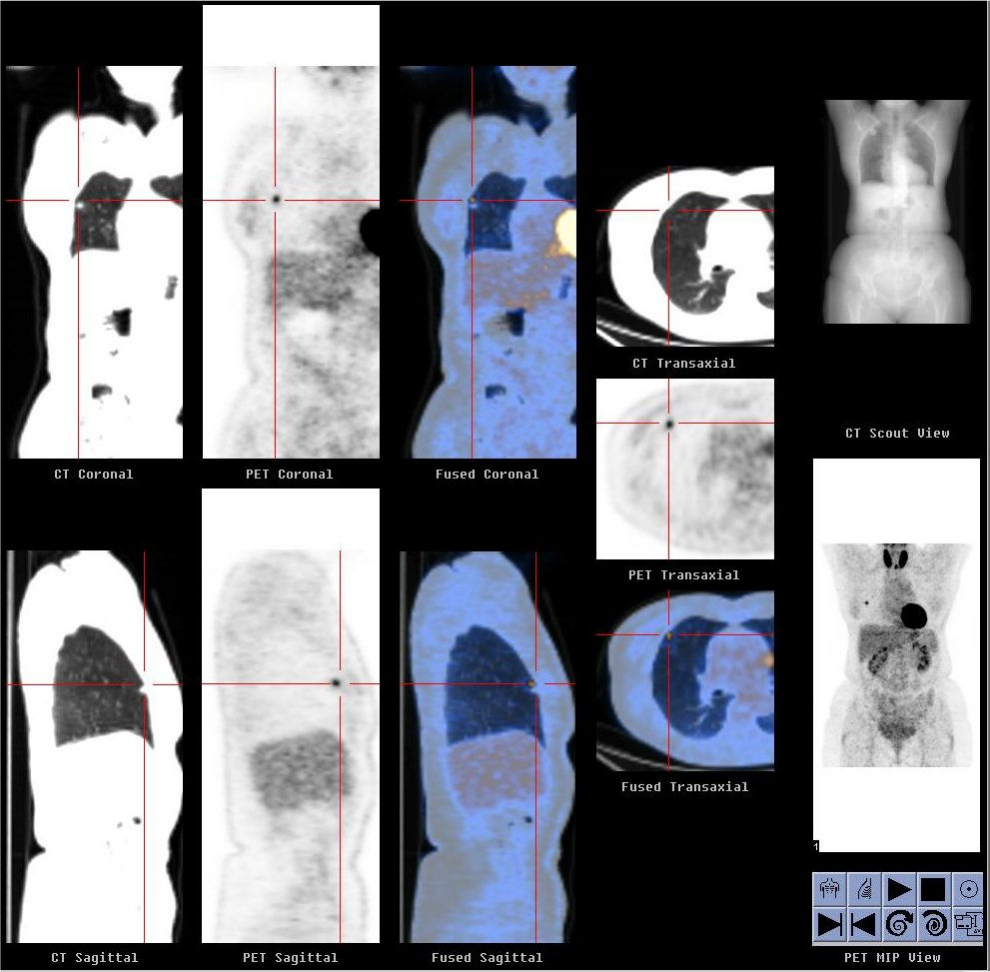
Misregistration of CT and PET malignant nodule of the right lung due to respiratory movement.

As described by Papathanassiou *et al*. [47], this phenomenon not only sometimes provokes misregistration of lesions between the two modalities (Figure 4) or disrupts image fusion of normal organs, but also may cause an erroneous attenuation correction. Because of respiratory motion the density of a particular organ could be attributed to an area whose density is different.

For example, the downward displacement of the diaphragm causes an underestimation of correction of the liver dome, leading to a cold area in that zone. It is obvious that particular attention is needed if the patient is suspected for liver metastasis or for nodules at the base of the lung.

The best way to correct for respiratory motion would be to acquire gated images to discriminate different intervals of a breath cycle. Many companies are working to implement hardware respiratory-motion correction on their systems, but none are currently completely validated.

## Truncation

The typical transverse field of view (FOV) of the CT scanner in a PET/CT system is about 50 cm, while the PET FOV is 70 cm. The relative small CT FOV can cause truncation of CT images [48]. To avoid truncation artefacts in PET/CT images patients are scanned with arms above their head. However, in obese patients and in scans acquired with arms down, as with some patients with melanoma or head and neck tumours, this kind of artefact is frequently seen.

As described by Mawlawi *et al.* [49] the aspect of truncation artefact in CT images is a bright rim of high attenuation values together with characteristic streaking, reflecting on PET-corrected images as absence of attenuation correction factors in the sections of the PET slices which exceed the CT FOV. The resultant artefact on the attenuation corrected PET images is an overestimation of the activity concentration corresponding to the rim and an underestimation corresponding to the region without attenuation factors.

Several techniques have been proposed and implemented on commercial systems to correct for truncation artefacts and most of them give a recovery of more than 90% of the activity in the truncated regions. Hsieh *et al* [50] developed an algorithm for truncation correction which extends the CT FOV based on information obtained from untruncated projections of the object and the knowledge that the total attenuation of an object should be the same independent of the projection angle. This technique has been implemented in the GE Discovery ST PET/CT system.

Although the different techniques are effective for normal size patients, images of large or obese patients need a deeper analysis and in all cases corrected SUV measurements must be used carefully.

## CONCLUSION

PET/CT improves quality accuracy of the image. The use of CT for attenuation correction yields several advantages in terms of accuracy and patient comfort.

Several artefacts are introduced in CTAC PET images due to CT, but their knowledge and the use of proper correction techniques, such as dedicated algorithms, which take into account the presence of high density materials, minimises any source of false findings.

To avoid possible pitfalls, the simultaneous display of both CTAC and non-corrected PET images, side by side with CT images is strongly recommended.
